# Perrogator: A Portable Energy-Efficient Interrogator for Dynamic Monitoring of Wavelength-Based Sensors in Wearable Applications

**DOI:** 10.3390/s19132962

**Published:** 2019-07-05

**Authors:** Camilo A. R. Diaz, Arnaldo Gomes Leal-Junior, Letícia M. Avellar, Paulo F. C. Antunes, Maria J. Pontes, Carlos A. Marques, Anselmo Frizera, Moisés R. N. Ribeiro

**Affiliations:** 1 Telecommunications Laboratory LABTEL, Electrical Engineering Department, Federal University of Espírito Santo, Espírito Santo 29075-910, Brazil; 2 Mechanical Engineering Department, Federal University of Espírito Santo, Espírito Santo 29075-910, Brazil; 3 Department of Physics and I3N, University of Aveiro, Campus Universitário de Santiago, 3810-193 Aveiro, Portugal; 4 Instituto de Telecomunicações, Campus Universitário de Santiago, 3810-193 Aveiro, Portugal

**Keywords:** fiber Bragg gratings, interrogators, Fabry-Perot interferometers, gait analysis, wearable applications

## Abstract

In this paper, we report the development of a portable energy-efficient interrogator (Perrogator) for wavelength-based optical sensors. The interrogator is based on a compact solution encompassing a white light source and the spectral convolution between the sensor and a tunable filter, which is acquired by a photodetector, where a microcontroller has two functions: (i) To control the filter tuning and to (ii) acquire the photodetector signal. Then, the data is sent to a single-board computer for further signal processing. Furthermore, the employed single-board computer has a Wi-Fi module, which can be used to send the sensors data to the cloud. The proposed approach resulted in an interrogator with a resolution as high as 3.82 pm (for 15.64 nm sweeping range) and maximum acquisition frequency of about 210 Hz (with lower resolution ~15.30 pm). Perrogator was compared with a commercial fiber Bragg grating (FBG) interrogator for strain measurements and good agreement between both devices was found (1.226 pm/µε for the commercial interrogator and 1.201 pm/µε for the proposed approach with root mean square error of 0.0144 and 0.0153, respectively), where the Perrogator has the additional advantages of lower cost, higher portability and lower energy consumption. In order to demonstrate such advantages in conjunction with the high acquisition frequency allowed us to demonstrate two wearable applications using the proposed interrogation device over FBG and Fabry-Perot interferometer (FPI) sensors. In the first application, an FBG-embedded smart textile for knee angle assessment was used to analyze the gait of a healthy person. Due to the capability of reconstructing the FBG spectra, it was possible to employ a technique based on the FBG wavelength shift and reflectivity to decouple the effects of the bending angle and axial strain on the FBG response. The measurement of the knee angle as well as the estimation of the angular and axial displacements on the grating that can be correlated to the variations of the knee center of rotation were performed. In the second application, a FPI was embedded in a chest band for simultaneous measurement of breath and heart rates, where good agreement (error below 5%) was found with the reference sensors in all analyzed cases.

## 1. Introduction

In the last few years, optical fiber sensors have experienced a popularity increase due to their advantages over conventional sensors technologies. These advantages include compactness, electromagnetic field immunity, chemical stability, multiplexing capabilities and passive operation [1]. Such advantageous features of optical fiber sensors have pushed the boundaries of optical fiber sensors instrumentation from the laboratory environment to field applications in diverse areas, such as industrial applications [2], structural health monitoring [3], medicine [4], robotics and healthcare [5]. 

The widespread of optical fiber sensors occurs in conjunction with the development of alternative interrogation techniques and new sensor approaches [6]. Nowadays, there are different alternatives in optical fiber sensor systems, where it is possible to develop solutions based on optical intensity variation [7], interferometry [8], nonlinear effects [9], fiber specklegram [10], surface plasmon resonance [11] and reflectometry using uniform [12] and non-uniform [13] fiber Bragg gratings (FBGs) and also long period gratings (LPGs) [14]. These different types of optical fiber sensors have been employed on the assessment of many physical and chemical parameters, where each sensor approach has its own advantages and drawbacks when compared with each other. As an example, intensity variation-based sensors have the advantages of allowing low cost solutions, portability, ease of signal processing and implementation, but handicapping sensitivity due to optical source power fluctuations that can result also in lower accuracy for the sensor [15]. On the other hand, sensors based on optical interferometers, LPGs and FBGs, the measurand is analyzed as a function of the wavelength variation. Thus, such sensors are insensitive to optical power source fluctuations [1]. In addition, they generally present higher accuracy and resolution than intensity-based sensors. Moreover, FBGs present the additional advantage of high multiplexing capabilities, where quasi-distributed sensor arrays can be inscribed at different Bragg wavelengths using only one fiber [16]. However, FBGs need specialized equipment for the grating inscription that involves expensive lasers and other optical apparatus, which results in a higher associated cost when compared with other optical fiber sensors such as Mach Zehnder and Fabry-Perot interferometers (MZI and FPI, respectively). They can be produced with simple and low cost methods, as summarized in [17] for FPIs. Nevertheless, optical interferometers do not have the multiplexing capabilities of FBGs. Thus, the sensors have to be wisely chosen according to the desired application.

For wavelength-based sensors, the wavelength-encoded data have to be extracted using interrogation devices such as optical spectrum analyzers (OSAs), spectrometers or any interrogation unit capable of reading the optical spectrum of the sensors. In general, these devices are either bulk (with poor portability) and expensive or have low acquisition frequency [18]. These characteristics of conventional interrogation systems are especially undesired for applications in which both portability and acquisition frequency are key factors. Aiming at tackling these limitations, different interrogation techniques for wavelength-based sensors (especially FBGs) have been proposed. These techniques are generally based on edge filters for FBGs, in order to transform the FBG’s spectral response into power variation [19]. Even though edge filtering was extensively investigated (e.g., using MZIs [20], LPGs [21], FBGs [22] and FPIs [19] as the edge filter element), such an interrogation method has the disadvantage of reduced multiplexing capabilities when considering applications with multiple embedded sensors. Optical time-domain [23] as well as optical frequency-domain reflectometry [24] are commonly employed for the interrogation of wavelength-based sensors but such techniques also result in a bulk system with low acquisition frequency, which is not suitable for dynamic measurements of embedded sensors in wearable devices.

Dynamic monitoring of human activities often has requirements that combined rule out conventional interrogation systems. In this particular application, portable systems with a high acquisition rate are mandatory. Monitoring human activities generally involve many physical parameters (such as joint angles, physiological parameters as well as the assessment of force and torque interactions) [25]. It is also worth noting that a multisensory low cost interrogation system is also highly desirable for such applications, where the user’s health condition or activities can be monitored from home as an e-health solution for patients monitoring [26]. Thus, low relative cost is also a requirement in wearable systems for healthcare or human activities monitoring. The compactness and flexibility of optical fiber sensors are very convenient features for healthcare and human activities monitoring, where sensors can be embedded in different materials or can be directly positioned on the human skin for joint angle [27] or physiological parameters [28] monitoring. In addition, the biocompatibility and electromagnetic field immunity of optical fibers result in additional advantages of these sensors when applications involving invasive monitoring, intense magnetic-field tomography, or wearable robotics are concerned. To that extent, wavelength-based sensors (such as FBGs, optical interferometers and LPGs) have been proposed (mostly using FBGs) on the assessment of joint angles [29], plantar pressure [30], arterial pulse [19], breathing and heart rates [31]. However, in these applications, commercial FBG interrogators were used, leading to a cumbersome device that reduces the sensor system’s portability [29]. Note that the application on arterial pulse monitoring uses the edge filter interrogation technique [19], thus only one FBG can be assessed with each filter and the FBG spectrum cannot be reconstructed, since only the correlation between the wavelength and optical power is analyzed. Thus, the edge-filter technique can result in information loss, especially for displacement (angle, strain or torsion) sensors, since it was demonstrated in [32] that the analysis of the whole FBG spectrum can provide additional information about the mechanical loadings on the grating, where it can be possible to obtain a 3D displacement sensor by this analysis.

This paper presents the development characterization and wearable applications of a portable interrogator for dynamic measurements. The proposed interrogator has two stages: (i) An optical stage that includes a tunable filter, light source, photodetector and optical circulator; (ii) an electronic stage with a microcontroller and small single-board computer (Raspberry pi 3), which already has an embedded Wi-Fi module, enabling remote health monitoring. The microcontroller tunes the filter in order to obtain a fast and high-resolution sweep on the filter optical spectrum and the convolution between the filter and the wavelength-based sensors under test is acquired by a photodetector for the spectral reconstruction, resulting in a system with lower cost when compared with commercially available interrogators. In order to show the feasibility of the proposed interrogation and fill a gap on realistic wavelength-based sensors wearable applications, we employed the interrogator on two study cases: (i) A knee joint angle (and axial displacement) assessment using FBGs; and (ii) a breathing (and heart) rate assessment using FPIs. Both applications require portability and a high acquisition rate from the interrogator. In addition, the knee joint assessment using FBGs require the reconstruction of the FBG spectra in order to obtain a simultaneous measurement of angle and axial displacement (using the technique discussed in [32]), where the spectral reconstruction is also important for FPI sensors if the analysis of each peak (or valley) is desired.

## 2. Materials and Methods

### 2.1. FBG Interrogator

**Figure 1:**
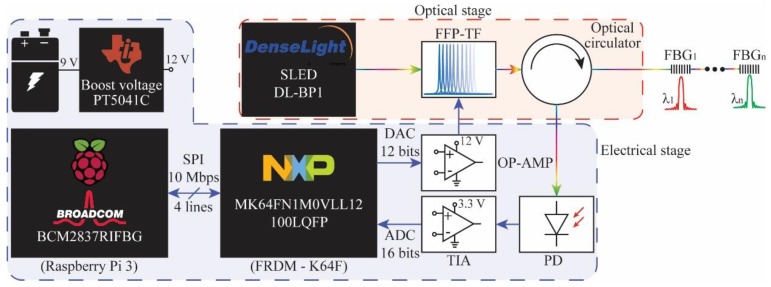
Schematic of the proposed interrogator, where the electrical and optical stages are in dashed and dashed-dotted lines, respectively. FBG: Fiber Bragg grating, FFP-TF: Fiber Fabry-Perot tunable filter, SLED: Superluminescent light-emitting diode, DAC: Digital-to-analog converter, ADC: Analog-to-digital converter, OP-AMP: Operational amplifier, PD: Photodetector, TIA: Transimpedance amplifier.

The FBG interrogator depicted in Figure 1 was divided into two stages. The optical stage (i) was composed by one fiber Fabry-Perot tunable filter (FFP-TF, Micron Optics, Atlanta, USA) with ~47 nm free spectral range (FSR) and first peak centered at ~1454.1 nm at room temperature ~23 °C. In addition, there was an optical circulator (Agiltron, Boston, MA, USA) and a superluminescent light-emitting diode (SLED) source (DL-BP1-1501A, Ibsen Photonics, Farum, Denmark) centered at 1550 nm with 70 nm 3 dB spectral width and 12 mW maximum optical power. The electrical stage (ii) was composed by a low-cost small single-board computer (SBC—Raspberry Pi 3, Cambridge, UK) with a quad-core ARM Cortex-A53 running up to 1.4 GHz central processor unit (CPU) and by a microcontroller unit (MCU) featuring an ARM^®^ Cortex^®^-M4 core running up to 120 MHz (Kinetis K64F, NXP Semiconductors, Eindhoven, Netherlands). The MCU controls the tunable filter and performs the signal acquisition with an on-chip digital-to-analog converter (DAC) with 12 bits resolution and analog-to-digital converter (ADC) with 16 bits resolution.

Since the DAC output value was from 0 V to 3.3 V, it was necessary to use an operational amplifier (OP-AMP, UA741C, STMicroelectronics, Geneva, Switzerland) to tune the FFP-TF wavelength over the whole FSR value (9.9 V ≈ 43 nm). When one value of the DAC was set, the digital-to-analog conversion was performed, i.e., per each DAC value there was one ADC value. After finishing the sweeping task, the raw data was transmitted from the MCU to the SBC. The communication between both modules was performed by serial peripheral interface (SPI) up to 10 Mbps with no package loss. The SPI is a synchronous serial communication interface composed by one Master (the SBC in this case) and several slaves (in this case, the MCU), which allows us to achieve high data transmission for short distances. In order to speed up memory operations on the MCU, the direct memory access (DMA) controller was used for the SPI communication. DMA allows the device to send or receive data directly to or from the main memory bypassing the CPU, resulting in better system performance. In the SBC, the SPI protocol and the data acquisition were programmed in C programming language. The main advantage of C programming is to access powerful low-level machine functions and execute the C code almost as fast as the assembly code, leading to low computational cost.

In the optical stage, the SLED optical source was launched to the FFP-TF, which performed the wavelength sweeping from ~1522 nm to ~1567 nm with 0.5 nm 3 dB bandwidth. The maximum resolution and sweep frequency at full scale were ~11.01 pm and ~26.65 Hz, respectively. These parameters could be modified leading to higher frequency rates depending on the quantity of points that were selected in the DAC. For instance, 2048 DAC steps lead to ~54 Hz frequency rate. However, there is a tradeoff between both parameters. Higher frequencies imply either less resolution or a less dynamic range. This feature results in an interrogator that is more adaptable to a given application. The FFP-TF output optical power was launched into the FBGs by using an optical circulator. Then, the FBG’s backscattered spectrum was detected by a photodetector (GT322D, Go4fiber, Hong Kong, China), which was amplified with a transimpedance amplifier (MCP6021, Microchip, AZ, USA) and posteriorly acquired by the ADC. A printed circuit board contains the OP-AMP, TIA, PD and pin headers to interconnect the SBC with the MCU.

**Figure 2:**
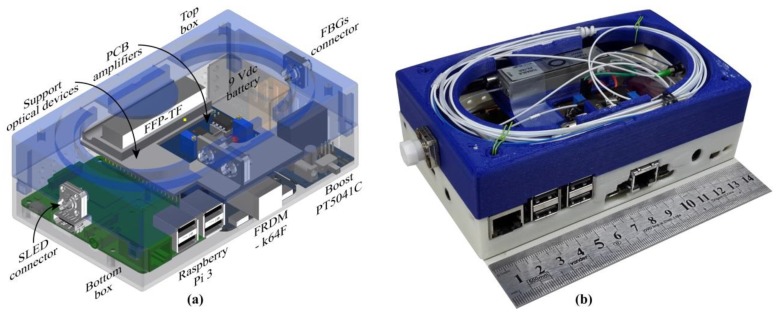
Representations of the portable interrogator: (**a**) 3D design and (**b**) picture of the portable interrogator.

In order to integrate all the components, an acrylonitrile butadiene styrene (ABS) box was 3D printed. The box model is depicted in Figure 2a, where it can be observed the internal distribution of electrical and optical components. The proposed structure was divided into two parts. The bottom part positioned all electronics devices and the top part housed all optical devices such as optical connectors, pigtails, FFP-TF and optical circulator. This configuration allows an easy integration between both electrical and optical stages. It is worth mentioning that the SLED was not incorporated in the Perrogator box, since it would increase the box size and would limit the capability of the proposed device of working with different light sources. Figure 2b shows a picture of the interrogator. The dimensions are 14.5 cm × 8.5 cm × 4.5 cm.

### 2.2. Caracterization and Validation of the Proposed Interrogator

The experimental characterization was performed in the electrical and optical stages. First, the capabilities of the MCU to generate the sweeping signal (triangular shape) to control the FFT-TF and the ADC conversions rate were analyzed in terms of samples acquisition frequency. In order to achieve the best performance, the MCU clock was set to a maximum frequency (120 MHz), and the ADC module was configured without hardware average and maximum clock speed. Since the SPI protocol has 8 bits, it is necessary to convert the ADC data from 16 bits to 2 bytes separately. DAC, ADC and adjusts of ADC values processes take ~7.56 µs per sample (~132 KHz). Therefore, a total sweeping period (ΔTs) takes 31 ms, which represents a baud rate of 132 kbps. The total raw data to be transferred after one ΔTs is a vector of 8192 bytes. The MCU used in SPI slave configuration implemented in this approach supports a maximum transfer rate of 15 Mbps. In order to avoid lost packages, the SPI master clock was configured as 10 Mbps. Therefore, the transfer process (ΔTx) takes ~6.6 ms at full-scale resolution. Since the MCU process is serial (instead of parallel), the total period (ΔT) to sweep and transfer the raw data is ~37.5 ms (~26.65 Hz). Table 1 summarizes different configurations of the proposed interrogator in terms of resolution and frequency rate, where the full-scale FFP-TF resolution is achieved by using the highest OP-AMP gain, leading to ~45 nm sweeping range. In the case of maximum FFP-TF resolution, the OP-AMP gain was set to 1, where the sweeping range was reduced to 15.64 nm and the achieved resolution was 3.82 pm. Therefore, the system resolution is sensitive to DAC resolution, number of DAC samples/points and OP-AMP gain, which can be tuned according to the measurement requirements. In addition, the frequency rate depends on the DAC resolution (12 bits or 4096 points) and the number of points, which can be easily configured from 512 points to 4096 points, leading to a faster or slower sweep on the optical spectrum.

The FFP-TF response was characterized with dynamic measurements under controlled temperature. A similar structure as presented in [18] based on Peltier and thermoelectric controller were used to kept the FFP-TF temperature constant. A periodical signal of ~13 Hz with ~9.5 Vpp was generated to feed the FFP-TF and a 17 KHz spectrometer (I-MON 512 HS, Ibsen Photonics) was used to monitor the temporal response of the filter in the optical domain. In the electrical domain, a 100 MHz bandwidth oscilloscope (MDO3012, Tektronix, OR, USA) allows us to monitor the signal that feeds the filter. To characterize the FFP-TF temperature sensitivity, the filter terminals were short-circuited to guarantee 0 V, and the temperature was increased from 25 °C to 45 °C in steps of 5 °C. Each step was increased after 30 min in order to wait the temperature stabilization. A commercial interrogator (SM125, Micron Optics, Atlanta, GA, USA) was used to monitor the FFP-TF spectrum.

The proposed interrogator response was characterized with static measurements under a controlled temperature, where an FBG sensor positioned as near as possible of the FFP-TF was used as a reference. In order to validate the proposed interrogator, a strain test was performed with another FBG. This FBG sensor was fixed with cyanoacrylate glue in its extremities between a rigid fixed platform and a linear axial translation stage. The selected distance between anchorage points was 240 mm. The strain characterization was performed at room temperature ~23 °C by increasing the strain response from 0 µε to 833.3 µε in steps of 83.3 µε. The backscattered spectra of both FBGs (reference and sensor) were monitored with a commercial interrogator (SM125) as a reference for the proposed approach.

In a portable system, power consumption is a critical parameter. Therefore, the total consumption of the system was analyzed. First, it was determined the maximum power required for the SLED to achieve a signal-to-noise radio higher than 20 dB. Then, the current consumption of the overall system was measured leading to ~615 mA. 

### 2.3. Wearable Application

In the wearable applications, the optical fiber sensors were embedded into two different flexible structures, namely a knee brace and a chest band for the knee angle and respiration assessments, respectively. Thus, the proposed sensors in conjunction with the proposed portable interrogator are well aligned with the requirement for smart textiles in healthcare applications. For this reason, an FBG is embedded in the knee brace, whereas the FPI is positioned in a chest band. As reported in previous work [33], it is possible to decouple the temperature cross-sensitivity of the sensors by using a FBG-based temperature sensor and applying the *K* matrix method. However, since the validation of the proposed approach is performed under controlled temperature, the influence of temperature can be neglected.

**Figure 3:**
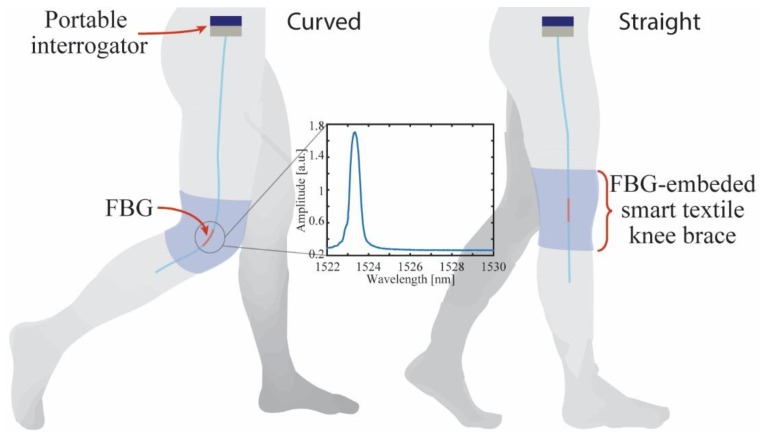
Schematic representation of the FBG-embedded smart textile for knee angle and displacement assessment. The inset shows the grating spectrum acquired by the portable interrogator.

The FBG-embedded smart textile for knee angle assessment is depicted in Figure 3, where the figure inset shows the FBG spectrum acquired by the portable interrogator. The FBG was inscribed in a photosensitive silica optical fiber using the phase mask technique with a KrF laser at 248 nm. The fiber has an acrylate protection in order to increase its mechanical robustness, especially for bending on the grating region. However, the knee is a polycentric joint and, as such, does not have a constant center of rotation. The model of the knee is a four-bar linkage, and this polycentric design allows knee stability to be varied throughout the gait cycle, increasing stability at heel-strike and reducing it at toe-off. An instantaneous center of rotation of the knee joint is posterior to the ground reaction vector when stability is required during loading and is anterior (unstable) during the late stance to allow knee flexion to be initiated [34]. Three-dimensional imaging such as magnetic resonance imaging and computer modeling have enabled great advances in research into knee kinematics, and have made possible the analysis of kinematics parallel to the planes of motion and incorporation of conjoint rotation. [35]. Despite the front of the knee has a larger displacement and response than the side of the knee (inducing maximum displacement of the FBG wavelength shift), the sensors used to measure angle (such as goniometers, encoders, IMUs and even the proposed sensors in this work—FBGs) are usually aligned with the joint in the sagittal plane since the sensor is located nearer of the knee joint center of rotation. On the other hand, when vision systems are used to analyze gait parameters, this plane represents the largest movement, which is a good reference to compare angle sensors. In addition, considering instrumentation in exoskeletons, for instance, the sagittal plane also is preferred since the mechanical structure is projected over this plane.

In order to obtain a two-dimensional representation of the knee joint displacements, we employ the technique proposed in [32], where the variations on the FBG reflectivity, wavelength shift and full width half maximum were used to decouple bending, torsion and uniaxial strain components on the FBG response. In the knee joint analysis, we used the reflectivity and wavelength shift data to decouple the axial and angular displacements on the knee during the gait. The first step for decoupling the axial and angular displacements is to characterize the sensor with respect to the individual contributions of each displacement condition. Thus, the FBG-embedded knee brace is positioned on a goniometer (with fixed center of rotation) for the angle characterization. Then, the sensor is positioned on a linear translation setup in order to obtain the axial displacement without bending or curvature. The FBG reflectivity variation and wavelength shift are analyzed for each case, resulting in linear regressions of these parameters as function of angle and uniaxial strain. Thereafter, it is possible to apply the coefficients obtained on the linear regression in Equation (1) to decouple the axial strain from the bending angle, where the coefficients matrix (*K*) is depicted in Equation (2). It is worth noting that the matrix *K* needs to be well-conditioned, i.e., det(*K*) ≠ 0, otherwise the technique for effects decouple is not applicable, since matrix *K* is not invertible when det(*K*) = 0.

(1) $$\left[\begin{array}{c} \varepsilon \\ \alpha \end{array}\right]={\left[\begin{array}{cc} s_{\lambda ,\varepsilon } & s_{r,\varepsilon }\\ s_{\lambda ,\alpha } & s_{r,\alpha } \end{array}\right]}^{-1}\left[\begin{array}{c} \Delta \lambda_{B}\\ \Delta r \end{array}\right]$$

(2) $$K={\left[\begin{array}{cc} s_{\lambda ,\varepsilon } & s_{r,\varepsilon }\\ s_{\lambda ,\alpha } & s_{r,\alpha } \end{array}\right]}^{-1}$$

where *ε* is the axial strain, *α* is the bending angle, Δ*λ_B_* is the wavelength shift and Δ*r* is the variation on the grating reflectivity. Moreover, *s_a,b_* are the coefficients obtained on the linear regression, where *a* is the spectral characteristic analyzed (*λ* for the wavelength and *r* for the reflectivity) and *b* is the parameter analyzed, i.e., axial strain and bending angle in this case.

**Figure 4:**
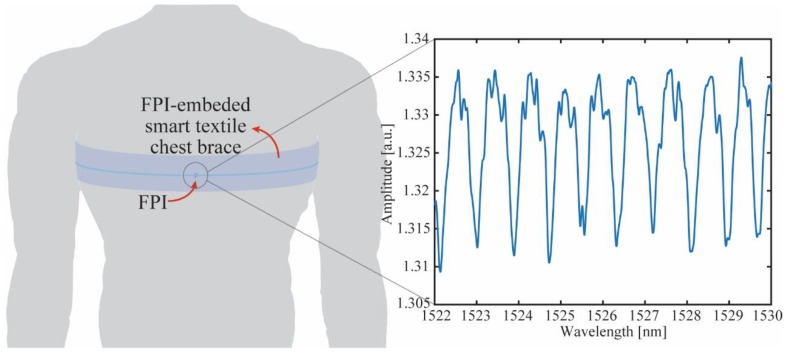
Schematic representation of the FPI-embedded smart textile for simultaneous measurement of breath and heart rates. The inset shows the FPI spectrum acquired by the portable interrogator.

For the physiological parameters analyses, breath and heart rates, an FPI was used due to its simplicity on fabrication and higher sensitivity when compared with FBGs. Another reason of using this sensor approach was to evaluate the proposed interrogator with a different wavelength-based sensor, instead of only evaluate the device as function of FBGs. In this case, the FPI cavity was produced using an ultraviolet (UV) curable resin in between two single mode silica fibers (SMF) as proposed in [36]. This technique leads to the production of FPIs in a simple and straightforward manner, where the sensitivity of the sensor can even be increased (or tuned) by adding additional layers of UV-curable resin [37]. For these reasons, we employed this technique on the FPIs production, where we first place two pieces of SMF-28 (Corning, New York, NY, USA) on a 3D translation stage with micrometer resolution for the fibers alignment. Then, a drop UV-curable resin Loctite AA 3936 (Henkel, Düsseldorf, Germany) was placed in the axial gap between the two fibers. Thereafter, a second alignment was performed, where axial displacement was made to define the cavity length by means of monitoring the FSR of the FPI on the interrogator. As the last step on the FPI production, an UV curing lamp was used (UTarget-365, AMS Technologies, Munich, Germany) with 1400 mW/cm^2^ radiance at 365 nm, which was focused on the FPI’s cavity with the UV-curable resin for about 40 s to promote the curing. The FPI spectrum is shown in the inset of Figure 4. 

After the FPI production, the sensor is positioned in the chest band as depicted in Figure 4, where the variations on the chest circumferences due to the respiration cause a curvature change on the FPI, resulting in a wavelength shift as discussed in [38]. It is worth noting that the breath leads to periodic variations on the peak wavelengths of the FPI. Similarly, the heartbeat also induces periodic vibrations on the FPI that can lead to a wavelength shift on its response. Although the breath-induced curvature variation has much higher amplitude than the ones associated with the heartbeat [39], such variations can be decoupled if the sensor analysis is performed in the frequency domain, since both signals have different frequencies (0.1 to 0.8 Hz for breath rate and 1.0 to 3.5 Hz for heart rate). Thus, the analysis is performed by means of applying the fast Fourier transform (FFT) in the sensor response. Then, the signal is filtered using a Butterworth filter in two different frequency windows, 0.1–0.8 Hz and 1.0–3.5 Hz, which results in the breath and heart rates as depicted in [28].

## 3. Results and Discussions

### 3.1. Portable Interrogator Characterization

**Figure 5:**
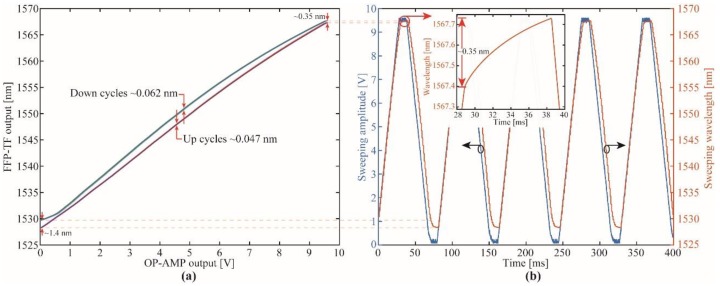
Characterization of the fiber Fabry-Perot tunable filter: (**a**) FTF-TF output as a function of the applied voltage for 11 cycles; (**b**) sweeping wavelength (measured with an Ibsen spectrometer) as a function of sweeping applied voltage (measured with oscilloscope).

The OP-AMP output as a function of the DAC output voltage was analyzed from ~0 V to ~3.3 V and back to ~0 V in steps of ~0.205 V. No hysteresis was found in both measured cycles (up and down). The non-inverting OP-AMP gain was approximately 2.878 in agreement with the projected value (~3) by using standard resistors with 5% tolerance. The FFP-TF output as a function of the applied voltage (OP-AMP output) is shown in Figure 5a. In order to verify the accuracy of the proposed approach, 11 continuous cycles of sweeping wavelength shift of both up and down cycles were analyzed. The mean standard deviation in the up cycle was ~0.047 nm, which represents a deviation of 0.122%. In the case of the down cycle, the standard deviation was ~0.062 nm, leading to a deviation of 0.165%. Since the FFP-TF is driven by a lead zirconate titanate (PZT)-element, it can be modeled as a capacitive load whether low frequencies are used to driven it. Due to the length cavity being changed with a PZT there is a phase delay between the electrical signal and the mechanical action, which depends on the sweep frequency. In addition, the displacement for a given voltage is not symmetric, which means the filter central wavelength peak is different in both cycles, i.e., increasing and decreasing sweeping voltages as it can be observed in Figure 5a. This nonlinearity can be compensated by either calibrating the filter on both up and down cycles separately or by using complex schemes of hysteresis linearization [40,41]. Furthermore, as the temperature increases, the average DC capacitance increases while the contact resistance decreases, leading to measurement errors [42]. Due to the temperature affects the piezoelectric properties, i.e., piezoelectric, elastic and dielectric coefficients, as well as the corresponding electromechanical coupling factors [43], it is expected the PZT material used to control the cavity filter presents an axial displacement when temperature increases because the thermal strain effect [44], generating a wavelength redshift of the FFP-TF spectrum. Since the FFP-TF presents an acceptable repeatability (±0.047 nm) and the linear regression for the up cycle shows a correlation coefficient (R^2^) higher than 0.997 with a sensitivity of ~4.452 nm/V, the sensor’s spectral signature can be estimated with this linear regression. Figure 5b shows the temporal sweeping wavelength shift of the FFP-TF as a function of the applied sweeping voltage, where it is evidenced the hysteresis of the filter. The periodic behavior of the signal, monitored with the spectrometer, shows the difference between the up and down cycles. In addition, due to the raw data transmitted between each cycle, the filter is fed with DC signals of either ~0.1 mV or ~9.5 V around 10 ms, where the inertia effect is more evident leading to a wavelength drift of ~0.35 nm (see inset on Figure 5b in the up cycle and 1.4 nm in the down cycle. With this information, it is possible to compensate measurement errors. 

**Figure 6:**
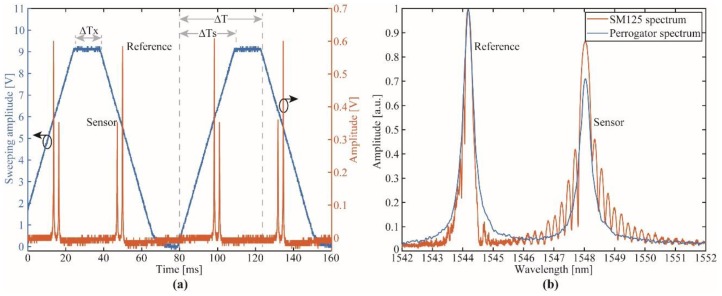
Spectral characterization of the FBG sensor: (**a**) Temporal response of the system; (**b**) optical spectra of both the SM125 interrogator and the proposed approach.

The temporal response of the proposed interrogator is shown in Figure 6a. According to the configurations of the SBC and MCU, it could be observed the ΔTs, ΔTx and the sum of both events ΔT. Superimposed to the FFP-TF control signal, it was presented the temporal response of the FBGs (reference and sensor). Since both FBGs have different physical length and consequently different reflectivity, it is evidenced in Figure 6a the maximum amplitude of the reference (~600 mV) was higher than the amplitude of the sensor (~350 mV). Therefore, the proposed approach was able to measure FBGs with high signal-to-noise radio (SNR) >25 dB (for a 5 mW BBS optical power). Nonlinearities occur at voltages lower than 1 V, limiting the use of the full FSR spectrum. Figure 6b depicts the spectra acquired with the SM125 interrogator and the proposed approach. As expected, the Perrogator spectrum presents a filtered version of the SM125 spectrum, since the step resolution is ~11 pm instead of 1 pm when compared with the SM125. In addition, the FFP-TF used in the Perrogator had a bandwidth of ~500 pm, which also limits the system resolution. 

**Figure 7:**
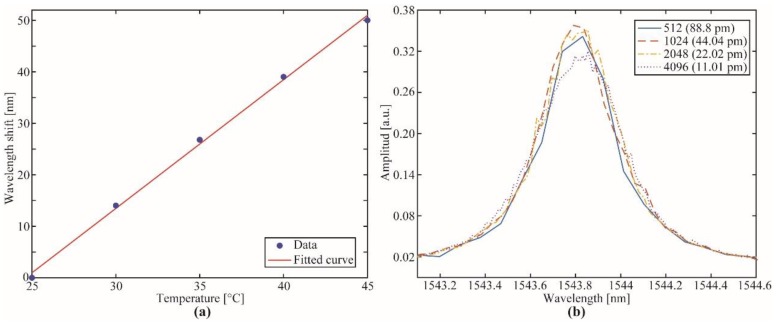
(**a**) FFP-TF wavelength shift as a function of temperature; (**b**) backscattered spectra of FBG as a function of FFP-TF resolution according to the DAC steps.

Figure 7a depicts the FFP-TF wavelength shift as a function of temperature for a fixed fed control signal (0 V). A sensitivity of 2.5 nm/°C was found, suggesting the use of the filter under controlled temperature. Figure 7 shows the backscattered spectra of FBG at different DAC steps resolution. In terms of bandwidth, no significant differences were observed in the measured spectra. This result could be attributed to the FFP-TF bandwidth, which was ~500 pm at 3 dB, and the lowest DAC resolution (512 steps) represented a 17% wavelength shift of the overall FFP-TF spectrum.

**Figure 8:**
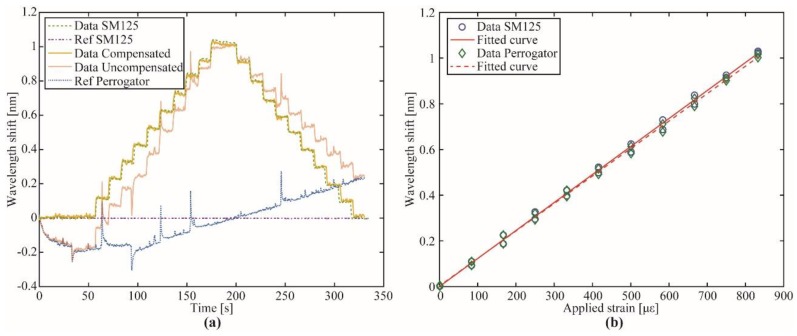
Strain characterization. (**a**) Compensation of temporal FFP-TF drift; (**b**) wavelength shift as a function of applied strain for both the SM125 interrogator and the proposed interrogator.

The temporal response of the applied strain is depicted in Figure 8a. The dashed and dashed-dotted curves represent the sensor and reference signals, respectively, acquired with the SM125 interrogator. Superimposed in solid lines are presented the compensated and uncompensated signals and the temporal drift error induced by the FFP-TF (dotted line) acquired by the Perrogator. By subtracting the reference signal from the sensor signal, it is possible to compensate errors induced by the FFP-TF. 

Figure 8b shows the strain characterization from 0 µε to 833 µε and back to 0 µε in steps of 83.33 µε. The solid line with circular markers represents the reference measure performed with the SM125 interrogator. Hysteresis around 0.037 nm was observed during the experiment, which represents a 3.65% error. According to the fitted curve, the sensitivity achieved was ~1.226 pm/µε with 0.998 correlation coefficient. The same experiment performed with the proposed approach is represented by the dashed line with diamond markers, where the strain sensitivity was very close to the reference value ~1.201 pm/µε with 0.998 correlation coefficient. However, the hysteresis was higher (~0.043 nm) leading to an error of ~4.23%. This value can be attributed to the filter hysteresis and manual error displacements in the strain platform. The mean standard deviation for the SM125 was ~0.0039 nm and for the compensated data of Perrogator was ~0.012 nm leading to an accuracy of ~12 pm.

Since the overall system was fed with a 10,000 mAh/37Wh power bank, it was expected an autonomy of 10 h, considering the consumption of both the SBC (~340 mA) and the MCU (~125 mA) at maximum performance, which results on a portable and energy-efficient interrogator. Several considerations are required in order to improve the first version of the portable interrogator. Temperature compensation is required in the FFP-TF in order to improve the accuracy of measures. In terms of hardware, more powerful SBC and MCU will improve the overall performance of the interrogator, since the ADC, DAC and SPI tasks will be executed faster. However, the battery autonomy will be compromised. In this first version, the FRDM-K64F development board version was used where no external peripheral modules were required. Therefore, all electronics devices can be integrated in a single PCB, leading to a small footprint. In terms of software, real time processing signal can be implemented on-chip or in the cloud since the SBC has on-board modules of communication Wi-Fi 2.4/5 GHz and Bluetooth low energy. In addition, the SLED optical source needs to be integrated to the interrogator, which can be easily accomplished by increasing the interrogator width in 4 cm. Since with 5 mW optical power it is possible to achieve a SNR higher than 25 dB, it is expected to use a 5 mW SLED with 14 pins butterfly package, where the TEC and current driver will be integrated in one small PCB (~40 × 40 mm, currently in development). Finally, there is a trade-off between autonomy and portability. According to the application, the power bank can be easily replaced by a smaller one sacrificing autonomy but reducing weight and size. 

### 3.2. Study Case 1: Knee Joint Assessment

**Figure 9:**
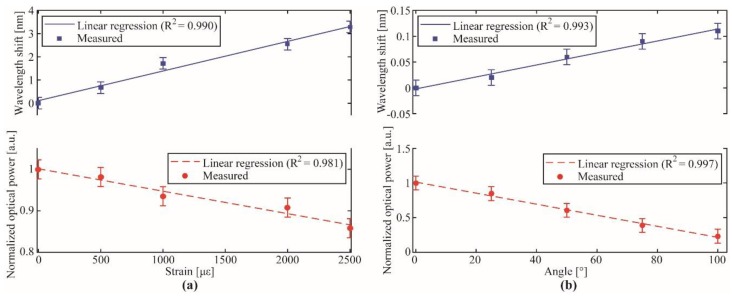
Characterization of the FBG-embedded smart textile for knee joint assessment: Wavelength shift and normalized optical power variation for the (**a**) strain response; (**b**) angle variation response. The error lines represent the standard deviation.

The characterization results for angle and axial strain variations for each spectral feature (reflectivity and wavelength shift) are presented in Figure 9a,b for axial strain and angle, respectively. The strain range tested was from 0 to 2500 με on steps of 500 με, whereas the angle was assessed on the range between 0 and 100° on 25° steps, which is within the knee range of movement during the gait [34]. Both experiments were performed three times, where no representative variation in the sensitivity was observed. Since, the characterization used a manual axial strain and angle setups, it is expected measurement errors (represented by the error lines). The mean standard deviation for wavelength shift and normalized optical power for strain characterization were 0.1 nm and 0.023 (dimensionless), and for angle characterization were 0.015 nm and 0.1 (dimensionless).

The results depicted in Figure 9 shows a higher wavelength shift variation with only minor reflectivity variations for the axial strain, whereas, for the angle analysis, the opposite occurs, i.e., there is a high reflectivity attenuation with low wavelength shift. Such results are in accordance with the ones simulated and experimentally demonstrated in [32]. From the linear regressions depicted in Figure 9a,b, it is possible to obtain the regression coefficients for each characteristic as function of the different displacement conditions analyzed as shown in Table 2. Considering the coefficient matrix presented in Equation (2), its matrix determinant (det(*K*); using the coefficients depicted Table 2) was about −0.01, which indicates that the technique for axial and bending displacements was applicable, since it was a well-conditioned matrix (det(*K*) ≠ 0).

**Figure 10:**
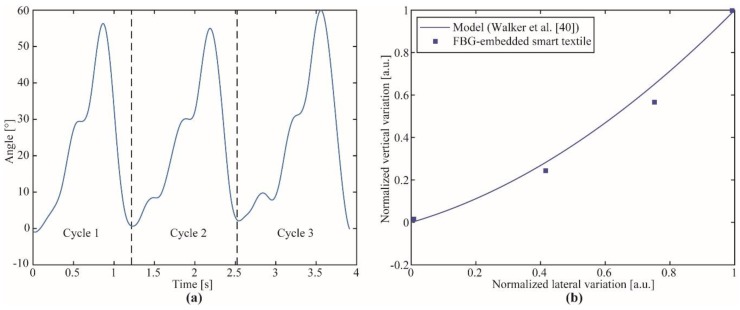
(**a**) Estimated angle using the FBG-embedded smart textile for gait tests. (**b**) Lateral and vertical displacements on the FBG for knee flexion cycles. The results are compared with the model presented in Walker et al. [45], which was derived from experimental measurements.

For the gait analysis, the knee brace with embedded FBGs was positioned on the right knee of a healthy subject without history of knee surgical interventions. The subject was asked to perform three steps with the velocity of his/her own choice, whereas the proposed interrogator acquires the sensor response. Then, the reflectivity and wavelength shift of the FBG were analyzed for each case, where Equations (1) and (2) were applied using the coefficients depicted in Table 2 in order to separate the angular and linear displacements on the sensor. Figure 10a shows the single-plane angular variation of the knee joint at each analyzed step, whereas Figure 10b shows the lateral and vertical displacements on the sensor on a flexion cycle of the knee. The vertical displacement was obtained from the axial strain on the FBG (using the technique for decoupling axial strain and bending). The lateral displacement was obtained using the measured bending angle, where the lateral displacement was estimated from trigonometric relations between the measured angle and the initial curvature radius (12.5 cm). 

The knee angle variation depicted in Figure 10a displayed a similar pattern as the one obtained in previous works using optical fiber sensors [29,46]. In addition, the angle range was within the range obtained from the knee joint for healthy subjects [34], where the maximum bending angle for the gait cycle was within the range of 50° to 60°. It is also worth noting that such angle variations from one cycle to another was within the range of standard deviations of the human movement [34]. Regarding to Figure 10b, the variations on the lateral and vertical displacements are in good agreement with the ones predicted by the model discussed in [45], which is a model widely adopted in the literature for the development of polycentric joints that resemble the knee movement or on wearable devices for the knee joint assistance [47]. The model proposed in Walker et al. [45] used as a comparison for the lateral and vertical displacements on the FBG-embedded smart textile was obtained as a regression from measured data using 22 subjects (14 ex vivo and eight in vivo) [45]. For this reason, it was used as a suitable reference for our analysis, which indicates that the proposed FBG sensor in conjunction with the portable interrogator could be employed not only on the knee angle assessment, but also could be a tool for the assessment of variations on knee center of rotation during the gait or dynamic activities. These advantageous features can aid on the assessment of gait-related pathologies as well as on the development and control of wearable assistive devices for the knee joint.

### 3.3. Study Case 2: Breath and Heart Rate Assessment

**Figure 11:**
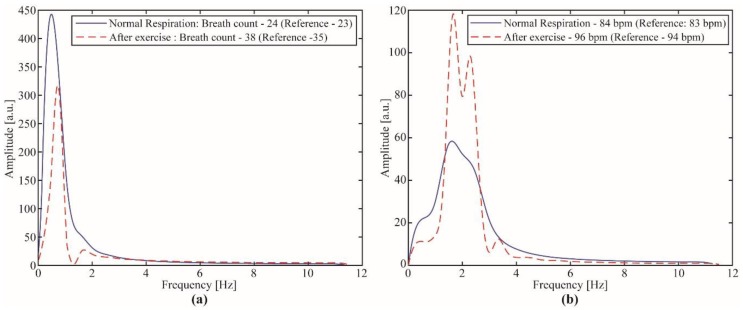
FPI-embedded smart textile for breath and heart rate responses under normal respiration and after exercise: (**a**) Breath count estimation (per minute); (**b**) heart rate estimation (in bpm—beats per minute).

For the breath and heart rates assessment, the FPI-embedded smart textile was positioned on the user’s chest and the signal was acquired for about 40 s. In order to analyze the sensor at different conditions, the FPI-embedded breath and heart rate sensor was analyzed in two conditions, one at which the user was under normal respiration condition and, the other after an event that resulted in higher respiration and heartbeat rates such as intensive exercises. The heart rate was compared with the one obtained from a reference sensor based on photoplethysmography (PPG; digiDoc Pulse Oximeter, Egersund, Norway), which provided a good accuracy as reported in [48]. The obtained results are depicted in Figure 11, where the sensor responses for the normal respiration and the respiration after intensive exercise in the frequency domain are displayed in Figure 11a,b for the breath and heart rate, respectively.

The results presented in Figure 11a show that the FPI-embedded sensor with the proposed interrogator was able to detect the breath with a high accuracy, where the error was higher for the case in which there was a high respiration rate. However, the highest error obtained was below 10% on the worst case, after an exercise where additional movements of the subject could lead to errors on the sensor breath estimation. Thus, the proposed sensor system (FPI sensor and portable interrogator) was a feasible option for non-invasive health monitoring with the possibility of remote monitoring using the Wi-Fi module of the interrogation device. Similarly, the heart rate assessment (performed after filtering the sensor response on the 1.0 Hz to 3.5 Hz frequency window) also showed good accuracy when compared with the reference sensor, where the maximum error was below 5%. The results of Figure 11 indicate the possibility of simultaneous assessment of breath and heart rates using the proposed approach (FPI-embedded smart textile) in conjunction with a portable interrogator with capabilities of high acquisition frequency (see Table 1).

### 3.4. Comparisons with Commercial Solutions and Future Perpectives

**Figure 12:**
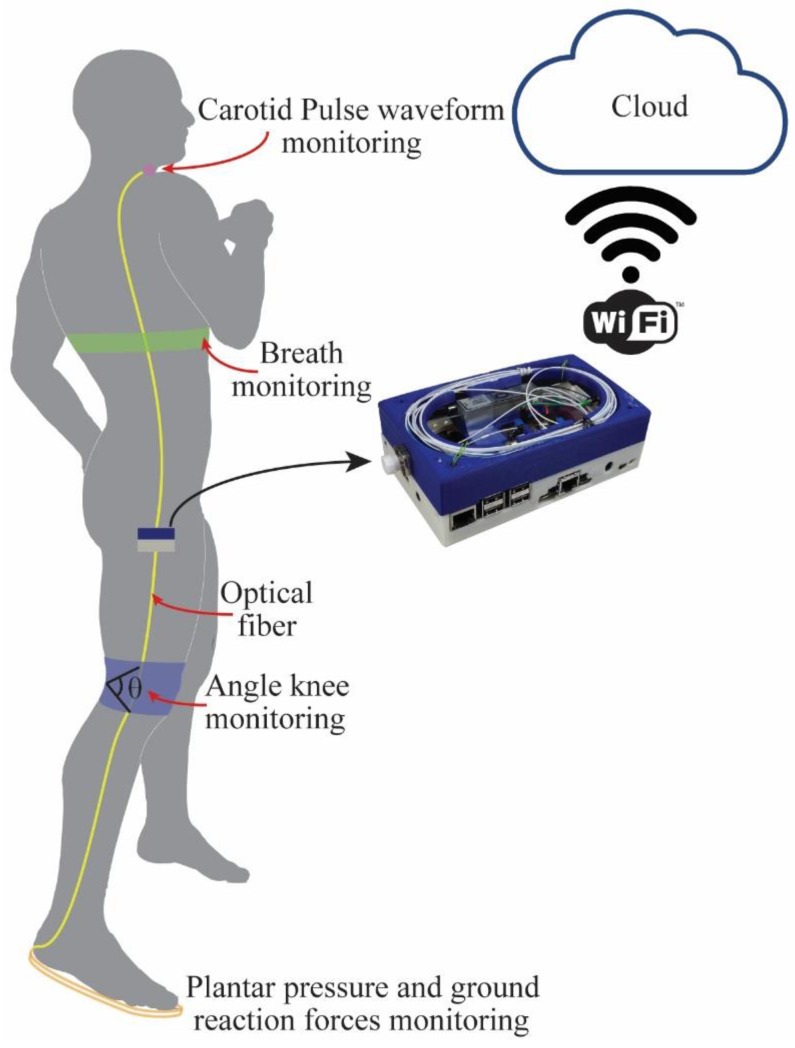
Schematic representation of the architecture for remote health monitoring using the proposed portable-energy efficient-interrogator.

The proposed interrogator has led to the possibility of portable and dynamic monitoring of different wavelength-based sensors, which indicates new possibilities of FBG applications on e-health assessment and remote health monitoring using fully portable devices. The FBG-embedded smart textile application also shows an interesting feature of the proposed interrogator, where it was possible to apply the technique for mechanical loadings decouple in FBGs proposed in [32] to obtain not only the angle variation, but also the vertical and lateral displacements on the FBG that can be correlated with the knee center of rotation. This result in a novel wearable sensor approach, where it was possible to fully address a polycentric joint (such as the knee) in order to completely describe its movement on the plane. Such assessment was only possible due to the advantageous features of the Perrogator, which enables the complete reconstruction of the FBG spectra in dynamic measurements that was used for decoupling axial and angular displacements. The tests using the FPI-embedded smart textile show the feasibility of the portable interrogator on the assessment of the dynamic parameters such as breath and heart rates, which are vital parameters for the human health assessment. In addition, it also shows the capability of the proposed device in reconstructing the spectra of a FPI under dynamic measurements, resulting in a novel optical interferometer-based sensor, where the curvatures on the FPI can be related to the respiration and heartbeat. Therefore, with the results reported in this work, one can propose an architecture for remote health monitoring using some of the optical fiber sensors for healthcare proposed in the literature, e.g., instrumented insoles, arterial pulse sensors, curvature sensors, breath and heart beat sensors as shown in Figure 12. These sensors are connected to the Perrogator, which is responsible for the signal processing (peak and power detection as well as simple operations such as the FFT) and the communication, where the data acquired from the sensor is sent to the cloud using the Wi-Fi module of the interrogator.

Table 3 presents a comparison between commercial interrogators and the proposed approach. Commercial interrogators have better performance in terms of accuracy and repeatability. However, the portability is the main limitation for wearable applications. In terms of portability, Perrogator offers a reduced size and weight (~300 g). In addition, the wireless communication feature simplifies its instrumentation. Another drawback for the commercial unit is the price, where the Perrogator offers the lowest price. In the next version, it is expected enhancing all the Perrogator parameters while keeping its lower price.

## 4. Conclusions

This paper presented the implementation and validation of a portable interrogator for dynamic assessment of wavelength-based sensors. The proposed interrogator is based on a tunable filter, where the convolution between the sensor and the filter spectra is acquired point-by-point by a photodetector. The filter is controlled by a microcontroller that is also responsible of acquiring the photodetector response, which is sent to a Raspberry Pi 3 for further signal processing and communication. The proposed approach resulted in an interrogator with a maximum resolution of 3.82 pm and acquisition frequency of up to 213 Hz. As an interesting feature of this interrogator, especially for portable applications, the expected autonomy was about 10 h, which enables a plethora of applications in different areas. The interrogator was validated with respect to a commercial FBG interrogator and good agreement between both devices was obtained. In order to present some of these many applications, the proposed portable-energy efficient-interrogator was employed on two wearable applications, namely knee angle and displacement assessment and simultaneous measurement of breath and heart rates. In these cases, FBG and FPI were embedded in different flexible structures in order to obtain smart textiles for the assessment of knee angle and respiration, respectively. The obtained results show the feasibility of the proposed interrogator and sensor systems, where the knee angle estimated was similar to the one obtained in previous works and the angles within the range measured for the knee joint [34]. In addition, the vertical and lateral displacement variations on the sensor during the flexion cycles were similar to the ones obtained in previous works, where the variation of the knee center of rotation was measured [45]. For the FPI-embedded smart textile for simultaneous measurement of breath and heart rates also resulted in good agreement between the sensor estimations and the reference sensors. Therefore, the good results obtained in conjunction with the demonstrated capabilities of the proposed interrogator open new avenues for portable and low-energy instrumentation using wavelength-based sensors, which can find major applications in e-health and remote health monitoring systems. Future works include tests on an architecture for remote health monitoring using the interrogator as well as further hardware improvements for an even higher autonomy, precision and acquisition frequency.

## Figures and Tables

**Table 1:** Comparison Between Resolution and Frequency Rate.

DAC Resolution	Full Scale FFP-TF Resolution (pm)	Maximum FFP-TF Resolution (pm)	Frequency Rate (Hz)
4096	11.01	3.82	26.65
2048	22.02	7.65	53.30
1024	44.04	11.47	106.61
512	88.08	15.30	213.22

**Table 2:** Coefficients of the sensitivity matrix obtained on the FBG characterization.

Parameter	Symbol	Value
Wavelength sensitivity—axial strain	*s_λ,ε_*	1.28 pm/με
Reflectivity sensitivity—axial strain	*s_r,ε_*	−5.14 × 10^−5^ με^−1^
Wavelength sensitivity—bending angle	*s_λ,α_*	1.16 pm/°
Reflectivity sensitivity—bending angle	*s_r,α_*	−7.98 × 10^−3^/°

**Table 3:** Comparative characteristics between commercial interrogators and Perrogator.

Interrogator/Parameter	SM125	I-MON 256 HS	Perrogator
Number of channels	4	1	1
Scan frequency (Hz)	2	17000	213
Accuracy (pm)	1	5	12
Repeatability (pm)	0.5	5	60
Dynamic range (dB)	50	30	25
Wavelength range (nm)	80	45	45
Battery powered	No	No	Yes
Wireless communication	No	No	Yes
Power consumption (W)	20	Not reported	3
